# Timing of repetition suppression of event‐related potentials to unattended objects

**DOI:** 10.1111/ejn.13972

**Published:** 2018-08-10

**Authors:** Gabor Stefanics, Jakob Heinzle, István Czigler, Elia Valentini, Klaas E. Stephan

**Affiliations:** ^1^ Translational Neuromodeling Unit (TNU) Institute for Biomedical Engineering University of Zurich & ETH Zurich Zurich Switzerland; ^2^ Laboratory for Social and Neural Systems Research Department of Economics University of Zurich Zurich Switzerland; ^3^ Institute of Cognitive Neuroscience and Psychology Research Center for Natural Sciences Hungarian Academy of Sciences Budapest Hungary; ^4^ Department of Psychology University of Essex Colchester UK; ^5^ Wellcome Trust Centre for Neuroimaging University College London London UK

**Keywords:** event‐related potential, general linear modeling, object recognition, predictive coding, repetition enhancement

## Abstract

Current theories of object perception emphasize the automatic nature of perceptual inference. Repetition suppression (RS), the successive decrease of brain responses to repeated stimuli, is thought to reflect the optimization of perceptual inference through neural plasticity. While functional imaging studies revealed brain regions that show suppressed responses to the repeated presentation of an object, little is known about the intra‐trial time course of repetition effects to everyday objects. Here, we used event‐related potentials (ERPs) to task‐irrelevant line‐drawn objects, while participants engaged in a distractor task. We quantified changes in ERPs over repetitions using three general linear models that modeled RS by an exponential, linear, or categorical “change detection” function in each subject. Our aim was to select the model with highest evidence and determine the within‐trial time‐course and scalp distribution of repetition effects using that model. Model comparison revealed the superiority of the exponential model indicating that repetition effects are observable for trials beyond the first repetition. Model parameter estimates revealed a sequence of RS effects in three time windows (86–140, 322–360, and 400–446 ms) and with occipital, temporoparietal, and frontotemporal distribution, respectively. An interval of repetition enhancement (RE) was also observed (320–340 ms) over occipitotemporal sensors. Our results show that automatic processing of task‐irrelevant objects involves multiple intervals of RS with distinct scalp topographies. These sequential intervals of RS and RE might reflect the short‐term plasticity required for optimization of perceptual inference and the associated changes in prediction errors and predictions, respectively, over stimulus repetitions during automatic object processing.

## Graphical abstract

We studied the time course of repetition effects on event related potentials (ERP) to objects. Model comparison favored an exponential model of repetition suppression—compared to linear or categorical “change detection”—indicating that repetition effects are observable in trials beyond the first repetition. We found successive intervals of repetition effects with distinct scalp distribution which might reflect the short‐term plasticity required for optimization of perceptual inference.

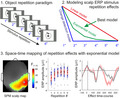

## 1 Introduction

Stimulus repetition‐related phenomena are ubiquitous in psychophysics, psychology, and neuroscience (Auksztulewicz & Friston, 2016; Barron, Garvert, & Behrens, 2016; Clifford et al., 2007; Grill‐Spector, Henson, & Martin, 2006; Henson, 2003; Ibbotson, 2005; Kohn, 2007; Krekelberg, Boynton, & van Wezel, 2006). Functional neuroimaging studies have observed both repetition suppression (RS) and repetition enhancement (RE) effects [reviewed in Segaert, Weber, de Lange, Petersson, and Hagoort (2013)]. RS, also referred to as stimulus specific adaptation (SSA), is thought to reflect a rapid form of experience‐dependent plasticity affecting perception and response properties of neurons. It has also been linked to optimization of the brain's predictions about the sensory environment (Solomon & Kohn, 2014) by discounting expected properties (Clifford et al., 2007; Summerfield, Trittschuh, Monti, Mesulam, & Egner, 2008; Vogels, 2016; Webster, 2011).

Repetition‐related phenomena in the visual system are complex and likely represent a compound of distinct neural processes, the mechanisms of which are not fully understood yet (Grill‐Spector et al., 2006; Ibbotson, 2005; Solomon & Kohn, 2014). At least three mechanisms contribute to SSA, including (a) somatic after hyperpolarization, (b) synaptic depression due to depletion of presynaptic vesicles, and (c) circuit‐level mechanisms (Kohn, 2007; von der Behrens, Bauerle, Kossl, & Gaese, 2009). Adaptation is thought to modify neural population coordination, and its effects cascade through the processing stages, possibly affecting multiple networks (Solomon & Kohn, 2014).

Predictive coding (PC) offers a neurobiologically plausible, mechanistic model for stimulus repetition‐related phenomena, and accommodates observations in psychophysics, electrophysiology, and functional neuroimaging (Auksztulewicz & Friston, 2016; Friston, 2005). PC suggests that the brain maintains and updates an internal model of the environment to infer the most likely causes of sensory inputs and actively generates predictions (Clark, 2015). In this framework, sensory systems are hierarchically organized where each level receives inputs from the level below that signals a mismatch between predicted and observed events, a prediction error (PE; Friston, 2005; Hinton, Dayan, Frey, & Neal, 1995; Hohwy, 2013; Rao & Ballard, 1999). In turn, each level sends its input to the level below conveying predictions that are thought to explain away PEs at the lower level. Perceptual inference, that is, the process of determining the most likely cause of sensory inputs, thus, rests on message passing across the hierarchy. From the perspective of PC, adaptation or RS can be understood as a neural correlate of perceptual learning, that is, the optimized process of perceptual inference where PEs to repeated stimuli are explained away more efficiently due to synaptic plasticity in sensory circuits (Baldeweg, 2007; Garrido et al., 2009). Conversely, RE could represent increased neural activity during the “sharpening” of predictions corresponding to increasing precision (implemented by increased postsynaptic gain) over repetitions. Encoding precision is important as veridical perception not only rests on the content of sensory signals but also on the confidence or precision of the signals that drive inference. It has been suggested that the brain might employ mechanisms to encode precision by relying on modulatory neurotransmitters that regulate the gain or excitability of populations (Friston, Brown, Siemerkus, & Stephan, 2016). Specifically, cholinergic mechanisms might affect the encoding of sensory precision by modulating postsynaptic gain (Feldman & Friston, 2010; Moran et al., 2013). Alternatively, cholinergic mechanisms can also affect the function of *N*‐methyl‐d‐aspartate (NMDA) receptors (Aramakis, Bandrowski, & Ashe, 1997; Chen et al., 2008) which, in turn, are thought be involved in signaling both PEs and predictions in cortical hierarchies (Corlett, Honey, Krystal, & Fletcher, 2011; Friston, 2005; Stephan, Diaconescu, & Iglesias, 2016). In sum, PC offers potential explanations for the phenomena observed during repeated stimulus presentation: larger responses to unpredicted events, and attenuation as well as enhancement to repeated events (de Gardelle, Waszczuk, Egner, & Summerfield, 2013; Egner, Monti, & Summerfield, 2010; Recasens, Leung, Grimm, Nowak, & Escera, 2015).

While studies on repetition of faces and words are relatively abundant (for a recent review, see Schweinberger & Neumann, 2016), there are few electroencephalography (EEG) studies where time‐course and topographic distribution of repetition effects to objects were investigated at the whole‐scalp level. Typically, most studies focused the analysis on a preselected set of electrodes and confined amplitude measurements to time windows either based on visual inspection of the data or on previous reports in the literature. Furthermore, previous studies often investigated brain responses only to the first repetition relative to the initial presentation, thus, ignoring the dynamics of brain responses to further repetitions (Andrade, Butler, Mercier, Molholm, & Foxe, 2015; Eddy, Schmid, & Holcomb, 2006; Gilbert, Gotts, Carver, & Martin, 2010; Gosling, Thoma, de Fockert, & Richardson‐Klavehn, 2016; Gruber, Giabbiconi, Trujillo‐Barreto, & Muller, 2006; Gruber & Muller, 2006; Guillaume et al., 2009; Henson, Rylands, Ross, Vuilleumeir, & Rugg, 2004; Kim, Jang, Che, Kim, & Im, 2012; Schendan & Kutas, 2003). The aim of the current study was to determine the time course and scalp distribution of repetition effects without prior hypotheses about the dynamics (RS vs. RE), time course, or scalp distribution of repetition effects. To this end, we analyzed the spatiotemporal dynamics of ERPs to black and white line drawings of common objects over six consecutive presentations. We used statistical parametric mapping (SPM; Friston, 2007) to analyze ERP amplitudes at each and every sensor in the poststimulus 50–500 ms time window using a mass‐univariate approach. Given that most studies focused on the analysis of ERPs to the first repetition only, little is known about the time course of the decay of scalp‐recorded ERPs to object stimuli that are repeated multiple times. Therefore, we set up three GLMs with parametric regressors incorporating three hypotheses about the time course of repetition effects. We used an exponential, a linear, and a “change detection” model to identify ERP components that showed a reliable repetition effect and performed Bayesian model comparison (Stephan, Penny, Daunizeau, Moran, & Friston, 2009) to identify the model that best explained the observed data.

## 2 Methods

### 2.1 Participants and ethics statement

Seventeen students (mean age 21.06 years, *SD* 1.56 years, 8 female, 15 right‐handed) volunteered to participate in the study. The experimental protocols were approved by the Institutional Review Board of the Institute for Psychology, Hungarian Academy of Sciences. All participants gave their written informed consent after the nature of the experiment had been fully explained. They received a monetary compensation for their participation in the study. All participants had normal or corrected‐to‐normal vision. The experiments were conducted in compliance with the Declaration of Helsinki.

### 2.2 Stimuli and procedure

**Figure 1:**
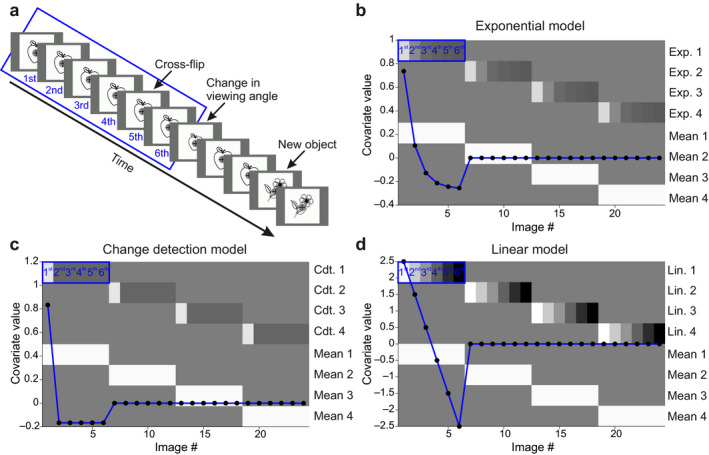
Paradigm and 1st‐level design matrix. (a) We used a simple stimulus repetition paradigm where line drawings of everyday objects were repeated 6–10 times. Between the 6th–10th presentations, a change in the viewing angle was introduced, after which the original picture was repeated two times. Note that our analysis focused on the first six presentations where stimuli did not change over repetitions. (b) Covariates plotted over the 1st‐level design matrix. Image number corresponds to images for mean event‐related potentials (ERPs) to the 1–6 presentations in four blocks (*x* axis). ERPs were modeled with a parametric modulator and a main regressor for each block (*y* axis right). The exponential function (mean centered) used for modeling repetition effects for the first block is plotted in blue over the design matrix (*y* axis left). (c and d) Covariates plotted over the 1st‐level design matrix for the change detection and the linear models, respectively.

In each of the four blocks, we recorded event‐related potentials (ERPs) to 60 black and white line drawings of common objects taken from the picture inventory by Szekely et al. (2004). The 60 object pictures were selected from the following semantic categories: small artifacts (*n* = 39, e.g., book, flag); large artifacts (*n* = 1, bed); objects found in nature (*n* = 2, flower, leaf); things to wear (*n* = 5, e.g., coat, hat); body parts (*n* = 2, feather, heart); foods (*n* = 11, e.g., apple, mushroom). Stimuli were organized into microsequences of 6–10 presentations of an object followed by a close‐up or wider‐angle view of the same object, and an additional repetition of the same object with its original viewing angle another two times (Figure 1a). ERPs to changes of viewing angle were not analyzed here. They were included to study boundary extension effects (e.g., Czigler, Intraub, & Stefanics, 2013) and will be published elsewhere. Thus, the length of microsequences varied pseudo‐randomly between 9 and 13 presentations. To study repetition effects, here, we analyzed ERPs to the first six stimuli, exclusively. In each block, we used 60 individual black line‐drawn objects on white background. Stimulus duration was 250 ms with 320 ms inter‐stimulus interval. Each picture subtended 11.4° visual angle and was presented on a dark gray background. A black fixation cross was presented in the center of the screen laid over a gray disk subtending 1.17° visual angle. To minimize eye‐movements, subjects were instructed to fixate at the cross throughout the experiment. Similar to our prior studies (Csukly, Stefanics, Komlosi, Czigler, & Czobor, 2013; Farkas, Stefanics, Marosi, & Csukly, 2015; Kovacs‐Balint, Stefanics, Trunk, & Hernadi, 2014; Stefanics & Czigler, 2012; Stefanics, Kimura, & Czigler, 2011), we employed a behavioral task to minimize the variation of attentional effects on the processing of object stimuli across participants by engaging the participants’ attention. Pseudo‐randomly every 3–6 s, the fixation cross became wider or longer (Figure 1a). The participants’ task was a speeded button‐press to the changes of the cross, and reaction time (RT) was recorded. Trials occurring within an 800‐ms interval after a change in the fixation cross were excluded from the analysis. RTs and hit rates were compared between experimental blocks with analyses of variance (ANOVA). Stimuli were presented using Cogent 2000 and Cogent Graphics developed at the Wellcome Department of Imaging Neuroscience (http://www.vislab.ucl.ac.uk/Cogent/index.html). Participants sat in a comfortable chair in a sound‐attenuated, dimly lit room during EEG recording.

### 2.3 EEG recordings and preprocessing

EEG was recorded from 61 Ag/AgCl electrodes according to a modified international 10–20 system (AF7, Fp1, Fpz, Fp2, AF8, AF3, AFz, AF4, F7, F5, F3, F1, Fz, F2, F4, F6, F8, FT7, FC5, FC3, FC1, FCz, FC2, FC4, FC6, FT8, T7, C5, C3, C1, Cz, C2, C4, C6, T8, TP7, CP5, CP3, CP1, CPz, CP2, CP4, CP6, TP8, P7, P5, P3, P1, Pz, P2, P4, P6, P8, PO7, PO3, POz, PO4, PO8, O1, Oz, O2). An electrode attached to the tip of the nose was used as reference. The ground electrode was placed on the forehead. EEG was recorded from DC with a low‐pass filter at 100 Hz (Neuroscan Synamp, Victoria, Australia). Eye movements were monitored by two horizontal and two vertical bipolar EOG electrodes. Data were digitized at 32 bit resolution and a sampling rate of 500 Hz. EEG was filtered off‐line between 1 and 30 Hz (24 dB/octave) and re‐referenced to the common average.

Epochs extending −100 ms before to 550 ms after stimulus onset were extracted from the continuous EEG for each object for the first six presentations. Epochs were baseline corrected to the pre‐stimulus 100‐ms period. To avoid other potential artefacts, epochs with values exceeding ±75 μV on any EEG or EOG channel were rejected from the analysis using the open source software EEGLAB (RRID: SCR_007292, Delorme & Makeig, 2004) in the Matlab development environment (MathWorks, Natick, USA). After artifact rejection, the total number of trials (summed over the four blocks) used for calculating the mean ERPs that entered the GLM was 197 (*SD *= 11), 184 (*SD *= 13), 183 (*SD *= 11), 182 (*SD *= 12), 187 (*SD *= 11), and 178 (*SD *= 11), for 1st, 2nd, 3rd, 4th, 5th, and 6th presentation of stimuli, respectively.

### 2.4 Space × time SPM analysis

Mean ERP data were converted to scalp ⨯ time images for statistical analysis using the open source software SPM12 (v6470, RRID: SCR_007037; Litvak et al., 2011), following similar preprocessing and statistical procedures as in previous work (e.g., Auksztulewicz & Friston, 2015; Garrido, Sahani, & Dolan, 2013; Henson, Mouchlianitis, Matthews, & Kouider, 2008; Stefanics, Heinzle, Horváth, & Stephan, 2018). The data were interpolated to create a 32 × 32 pixel scalp map for each time‐point in the poststimulus 50–500‐ms interval. Given a sampling frequency of 500 Hz, the time dimension consisted of 226 samples in each averaged ERP. Images were stacked to create a 3D space‐time image volume that was smoothed with a Gaussian kernel (full‐width at half‐maximum = [16 mm 16 mm 16 ms]) in accordance with the assumptions of Random Field Theory (Kiebel & Friston, 2004; Worsley & Friston, 1995).

A GLM with four main regressors corresponding to the four experimental blocks, and a generic decay/rise function as a parametric modulator for each stimulus presentation in each block, was estimated for each participant. The design matrix for a single subject is depicted in Figure 1b. The decay/rise function was used to quantify changes in ERP amplitude over repetitions within microsequences as unpredicted firstly presented objects became more predictable over consecutive repetitions. We chose an exponential function as parametric modulator as several electrophysiological and neuroimaging findings indicating that response attenuation over repeated stimulus presentations typically follows a non‐linear decay that is well approximated by an exponential function (e.g., Baldeweg, 2006; Boehnke et al., 2011; de Gardelle et al., 2013; Kaliukhovich & Vogels, 2014; Puce, Allison, & McCarthy, 1999; Sanchez‐Vives, Nowak, & McCormick, 2000). This allowed us to estimate modulation of the repetition sequence with an exponential time course. Importantly this design is flexible enough to capture both decay (attenuation or suppression) and rise (facilitation or enhancement) processes. For example, a decrease of a positive ERP component would show a positive correlation with our regressor across repetitions, whereas an increase of a positive component would show a negative correlation with our regressor (conversely for negative components). The estimated regression coefficients (beta parameter estimates) of the decay function for each scalp time‐point for each participant were analyzed at the group level for all three models (exponential, linear, “change detection”), using a standard two‐stage summary statistics approach (Friston, Stephan, Lund, Morcom, & Kiebel, 2005; Mumford & Nichols, 2009). These parameter estimates represent the relationship (similarity) between the dynamics of ERPs over repeated stimulus presentations and the parametric decay regressors.

On the group level, we used F‐tests to find scalp time‐points where mean ERPs were significantly modulated by repeated stimulus presentations. The resulting statistical parametric maps (SPMs) were family‐wise error (FWE) corrected for multiple comparisons at the voxel level (*p* < 0.05 [FWE]) using Random Field Theory (Flandin & Friston, 2017).

### 2.5 Model comparison

Beside using an exponential function as parametric modulator, we explored two alternative models previously considered for studying repetition effects (Lieder, Daunizeau, Garrido, Friston, & Stephan, 2013; Noppeney & Penny, 2006) and compared them with the exponential model. In particular, we considered repetition effects as (a) categorical decay (i.e., object 1st presentation > presentations 2–6) and (b) linear decrease. The categorical decay corresponds to a “change detection” model, that is, it represents the hypothesis that whenever a new object is presented in a sequence, it elicits a phasic response which disappears completely under the following repetitions. While the linear model is not realistic (given that RS effects must become consecutively smaller in physiological systems with decay mechanisms), we included this regressor as a “null” model, similar to Noppeney and Penny (2006). Thus, we set up another two GLMs, incorporating these two hypotheses. The design matrices for a single subject are depicted in Figure 1c,d, respectively.

To compare the models formally, we used the Bayesian Information Criterion (Schwarz, 1978) approximation to the log model evidence (LME). Under Gaussian noise (as assumed by the GLM), this leads to an approximation of LME that is a function of the residual sum of squares (RSSs):

(1) $$\text{LME}≃-\frac{1}{2}n\ln\left(\frac{\text{RSS}}{n}\right)-\frac{1}{2}k\ln\;\left(n\right)$$

where *n* is the number of data points and *k* is the number of parameters estimated by the model.

We first computed the LME for each voxel in individual participants. To perform model comparison at the group level, we computed the sum of ΔLME (between models) across subjects for each voxel. This is equal to the logarithm of the group Bayes factor (Stephan, Weiskopf, Drysdale, Robinson, & Friston, 2007) and corresponds to a fixed‐effects group‐level Bayesian model selection (Stephan et al., 2009) procedure. Group model comparison was performed both within a functionally defined mask (of voxels showing repetition effects under all models) as well as on all voxels in the 3D space‐time image volume (to perform an unconstrained comparison). The mask comprised all voxels from the SPM analyses where all three models had yielded a significant whole‐brain corrected effect (logical “AND” conjunction). We then used a non‐parametric Wilcoxon signed‐rank test to assess the null hypothesis of zero median for ΔLME across all voxels.

## 3 Results

### 3.1 Reaction time and hit rate

We compared RTs and hit rates for the occasional changes in the fixation cross between experimental blocks. An ANOVA of RTs across the four blocks yielded no significant effect (*F*_3,48_ = 1.12, *p* = 0.35) indicating lack of evidence for a change in reaction speed across the blocks. The mean RT was 452 ms (*SD *= 87 ms). An ANOVA of hit rate across the four blocks yielded no significant effect (*F*_3,48_ = 0.396, *p* = 0.76) indicating a high detection performance without signs of a change in hit rate during the experiment. The mean hit rate was 95.26% (*SD *= 6.7%).

### 3.2 Model comparison

**Figure 2:**
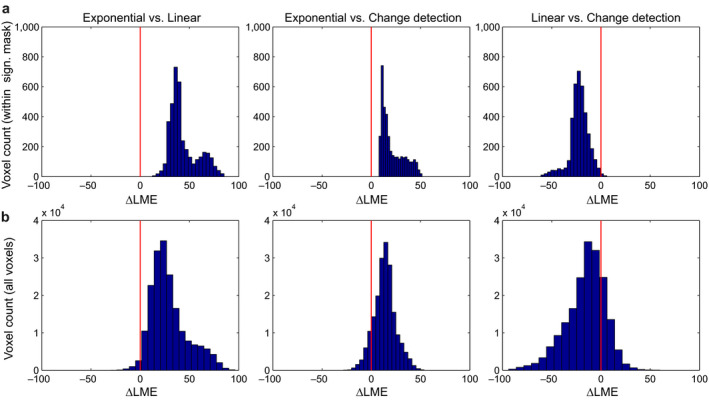
Histograms of ΔLME. (a) Histograms over the voxels within a mask defined by the “logical AND” conjunction of significant voxels under any of the three models, and (b) over all voxels in the whole 3D space‐time volume. LME: log model evidence.

We assessed the three models by performing model comparison at the group level as described above. The functionally defined mask was a conjoint mask of voxels showing significant repetition effects under any of the three models (logical “AND” conjunction). Fixed‐effects Bayesian model comparison revealed that the Exponential model was clearly superior compared to the other two models and that the Change detection model performed better than the Linear model, both for voxels within the functional mask (Figure 2a), as well as for voxels within the whole volume (Figure 2b).

To characterize the distribution of ΔLME values more formally, we performed null hypothesis testing, as described above. The results are summarized in Table 1.

**Table 1:** Summary of model comparison statistics. The results show that the distribution of ΔLME values is not Gaussian and median ΔLME values are significantly different from zero for all comparisons. The absolute value of median ΔLME in all comparisons was >12. Notably, a difference in LME >5 is considered as very strong evidence in favor of the superior model (Kass & Raftery, 1995)

ΔLME	Median	Mean	Std	% of voxels >0	Kolmogorov–Smirnov test (*D*)	Kolmogorov–Smirnov test (*p*)	Wilcoxon signed‐rank test (*Z*)	Wilcoxon signed‐rank test (*p*)
EXP vs. LIN (within mask)	38.77	43.59	14.24	100	1	<0.00001	−53.58	<0.00001
EXP vs. CDT (within mask)	16.73	21.48	11.42	100	1	<0.00001	−53.58	<0.00001
CDT vs. LIN (within mask)	−21.76	−22.11	9.63	0.57	0.9839	<0.00001	−53.57	<0.00001
EXP vs. LIN (all voxels)	25.03	29.15	18.58	98.36	0.9704	<0.00001	−375.75	<0.00001
EXP vs. CDT (all voxels)	13.78	13.73	11.03	89.28	0.8439	<0.00001	−348.01	<0.00001
CDT vs. LIN (all voxels)	−12.77	−15.43	19.98	21.43	0.7286	<0.00001	−283.30	<0.00001

*Note*

LME: log model evidence.

### 3.3 ERP results

**Figure 3:**
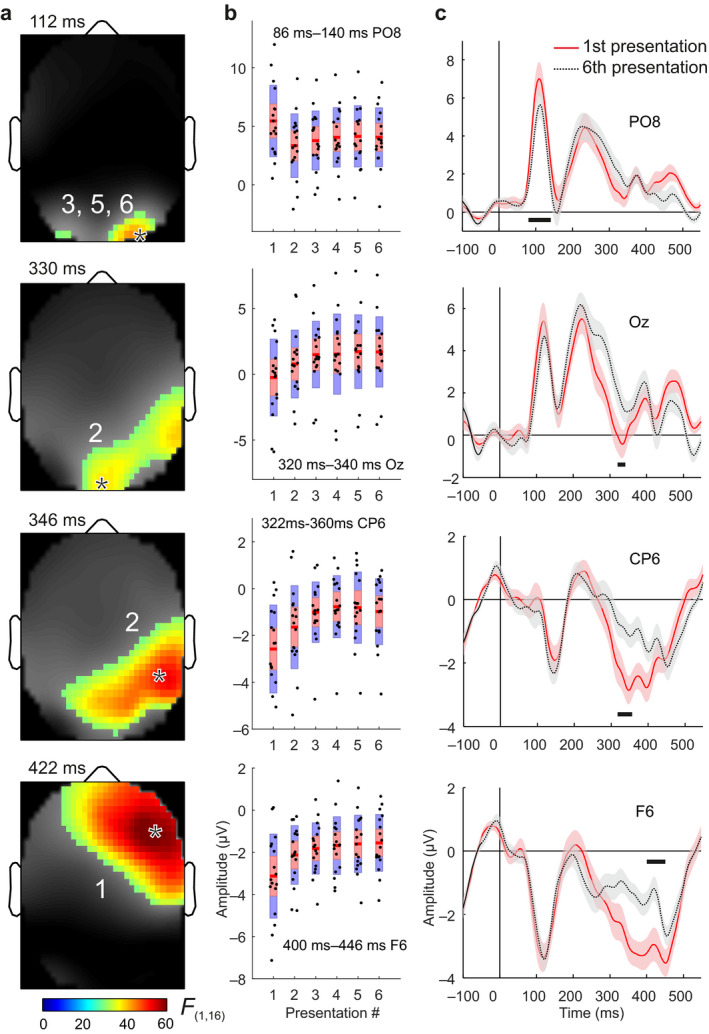
Statistical parametric maps (SPMs) and time course of repetition effects. (a) Main effect of stimulus repetition (pooled across the four experimental blocks), *F*‐values thresholded at *p* < 0.05 family‐wise error (whole‐scalp corrected), overlaid on the contrast images. Asterisks mark scalp locations for electrodes shown in (b) and (c). Numbers show activations as indicated in Table 2. Panels from top to bottom show observed intervals of repetition suppression (RS) in the 86–140 ms, repetition enhancement (RE) in the 320–340 ms, and RS in the 322–360 ms and 400–446 ms time windows, with occipital, occipitotemporal, temporoparietal, and frontotemporal distribution, respectively. Note that activation #4 is not plotted separately as it showed a similar dynamics and temporal topography to that of activation #2 peaking at 346 ms. (b) Box plots of event‐related potential (ERP) amplitudes for each stimulus presentation. Red lines represent the mean; points (subjects) shown together with the 95% confidence interval of the mean (1.96 *SEM*) in red and a 1 *SD* interval in blue. Time windows of significant repetition effects are indicated in each subplot at the electrode sites closest to corresponding cluster maxima. (c) Grand mean ERP waveforms elicited by the 1st and 6th stimulus presentation with 95% confidence interval are shown for illustration purposes. Note that statistics were carried out on 3D scalp space‐time parameter estimates which were based on ERPs for the 1st, 2nd, 3rd, 4th, 5th, and 6th stimulus presentation. Black horizontal bars mark intervals of significant repetition effects.

Given that model comparison showed that the GLM with the exponential decay function explains the data best, we used the space × time clusters with significant results for the winning model to illustrate repetition effects. We start from scalp topographies and then show using time‐windowed data and conventional ERP plots of the effects that lead to significant results in SPM. Scalp topographies of the SPMs are shown in Figure 3a for the cluster maxima. Model estimates to responses to line drawings showed a sequence of RS effects. Over repeated presentations of our visual stimuli, brain responses showed a sequence of RS and RE effects. An early RS effect was observed at bilateral occipital areas in the 86–140‐ms interval followed by RE at midline occipital sites (320–340 ms). Temporoparietal electrodes over the right hemisphere showed RS in the 322–360 ms time window followed by a further interval of RS in the 400–446 ms time window at right frontotemporal sites. Details of test statistics are given in Table 2. A video animation of the time course of repetition effects over the whole scalp is available in the Supporting Information of the online version of this paper.

**Table 2:** Test statistics for repetition effects. Significant activations are arranged and numbered according to size. *p*‐Values and statistics are given for up to three peaks within each activation

Activation number	Activation size (# voxels)	Latency (ms)	*p*‐Value (FWE‐corr.)	*F*‐statistic	Equivalent *Z*‐statistic
1	7,795	422	0.00180	61.2678	4.81524
		440	0.00404	52.1248	4.60680
		380	0.03835	31.7057	3.95946
2	4,156	346	0.00363	53.2627	4.63474
		346	0.00737	46.0016	4.44467
3	570	102	0.00474	50.4278	4.56392
4	52	298	0.03762	31.8552	3.96560
5	8	112	0.04366	30.7134	3.91801
6	2	108	0.04893	29.8551	3.88109

*Note*

FWE: family‐wise error.

For visual comparison, ERP amplitudes for each subject are shown in Figure 3b for each of the six stimulus presentations at selected electrodes in the intervals where significant repetition effects were observed. The early RS effect over occipital areas suggests an abrupt suppression of PE responses from the first to the second stimulus presentation, with constant response amplitude for further repetitions. In turn, the subsequent RS effects over temporal and frontal areas show a more smoothly decaying response profile. The occipital RE effect also shows a smooth time course of incrementally increasing response amplitude to repeated stimulus presentations.

For visualization, grand‐average ERP waveforms to the first and sixth stimulus presentations are shown in Figure 3c at electrode sites where SPM analysis had shown significant repetition effects. Our visual stimuli (line drawings of objects) evoked the canonical posterior P1 and P2 components, as well as prominent negative‐going peaks at parietotemporal and frontal electrodes. Note that ERPs are shown for illustration purpose only and no further null hypothesis tests are conducted; all statistical tests were carried out as part of the SPM analysis covering the entire space‐time volume, comprising data from all electrodes in the poststimulus 50–500‐ms interval (see Methods for details).

## 4 Discussion

In this study, we used SPM, a comprehensive analysis framework to investigate effects of stimulus repetition on ERPs to line drawings of everyday objects. For each subject, we estimated a GLM including a decay function to quantify changes in brain potentials over the whole scalp in the poststimulus 50–500 ms time window. We observed three consecutive intervals of RS in the 86–140, 322–360, and 400–446 ms time window with occipital, temporoparietal, and frontotemporal distribution (Figure 3a), respectively. Furthermore, we found an interval of RE in the 320–340 ms window with occipitotemporal distribution.

Importantly, similar to previous studies (e.g., Csukly et al., 2013; Farkas et al., 2015; Kovacs‐Balint et al., 2014; Stefanics & Czigler, 2012; Stefanics et al., 2011, 2018), our experimental design implemented a primary task to control attentional effects that might modulate repetition‐related neural activity (Auksztulewicz & Friston, 2016; Chennu et al., 2013, 2016; Egner et al., 2010; Gosling et al., 2016; Vuilleumier, Schwartz, Duhoux, Dolan, & Driver, 2005). The constantly high hit rate and fast RT over the experiment indicated that participants complied with the task and attended the fixation cross. This suggests that repetition effects observed in our study likely reflect automatic perceptual inference operating outside the focus of visual attention. Theories of perception as unconscious inference originate from Helmholtz's classical idea that perceptual experience is the “conclusion” of unconscious inductive inference from sensory input (Hatfield, 2002; Kiefer, 2017). In current theories of cortical information processing such as PC (Friston, 2005), RS is viewed as the result of a process during which the brain minimizes the PE (the difference between the predicted and the actual input) with increasing efficacy (due to updating of predictions and the associated synaptic plasticity of cortical connections) during repeated presentations of the same stimulus type. This decrease of PEs during processing of a given stimulus, and the change in efficacy of stimulus processing via plasticity in underlying neural circuits, corresponds to perceptual inference and learning, respectively (Baldeweg, 2007).

Our findings represent an important advance in the understanding of the time course and scalp distribution of repetition effects in ERP correlates of object perception as they are based on an unbiased mass‐univariate statistical approach that covers the entire space × time volume of EEG signals. Instead of relying on visual inspection and preselection of a subset of channels for amplitude tests, this approach included data from all electrodes and all time‐points in the poststimulus 50–500‐ms interval. The analysis takes into account the smoothness of scalp potentials (correlations between neighboring channels and temporal smoothness of the EEG signal) and used a stringent method for correcting for multiple comparisons (FWE) using Random Field Theory.

Repetition suppression has been widely used in fMRI studies that examine how specific representations may be encoded by neuronal population activity (e.g., Barron et al., 2016). Furthermore, models of network mechanisms have been put forward to explain RS at the level of population dynamics (Grill‐Spector et al., 2006). In electrophysiology, RS effects have been studied frequently over trials; by contrast, their temporal evolution within trials has received little attention. A PC view suggests that RS reflects the minimization of PE by updating predictions about the content and precision of sensory inputs (Auksztulewicz & Friston, 2015). Importantly, PC also suggests that for non‐trivial stimuli, which are processed at several (spatial, temporal, or semantic) scales, multiple significant within‐trial intervals of RS should occur. This is because model updating and the “explaining away” of PEs during perceptual inference occurs at several levels of the cortical hierarchy, with temporal delays inherent to the recurrent message passing between areas, and increasing synaptic efficacy due to short‐term plasticity is found on all levels (Baldeweg, 2006, 2007; Friston, 2005; Garrido et al., 2009). Our findings suggest that, for the particular stimuli used here (line drawings of everyday objects), this implicit perceptual process might take place in neural circuitry comprising occipital, temporal, and frontal areas.

Attention is known to counteract RS, that is, it increases neural response to the repeated stimuli (e.g., Auksztulewicz & Friston, 2015; Kok, Rahnev, Jehee, Lau, & de Lange, 2012). However, in our experiment, the object stimuli were task‐irrelevant and our protocol employed a primary task to engage the participants’ attention. Therefore, attentional effects are unlikely to account for the enhanced responses we observed in the 320–340 ms window with occipitotemporal distribution. RE has received less consideration than RS thus far (Segaert et al., 2013). RE effects in studies with implicit tasks have been suggested to reflect perceptual identification (Schnyer, Ryan, Trouard, & Forster, 2002) and involvement of explicit memory processes (Segaert et al., 2013). The PC theory assumes that activity of a subset of neural elements that participate in perceptual inference represent predictions about the content and precision of sensory inputs. During stimulus repetition, neural activity underlying both kinds of predictions might increase and manifest as enhanced neural responses. A prior study using fMRI reported that RS and RE co‐occurred in a single cortical region during stimulus repetition (de Gardelle et al., 2013). While our present analyses do not link our scalp ERP results to the source level, our findings of an early interval of RS in the 86–140 ms time window followed by an interval of RE in the 320–340 ms window with posterior distributions suggest occipitotemporal generator sources. Repetition paradigms with masked object (Eddy et al., 2006) and face (Henson et al., 2008) stimuli, as well an auditory study by Recasens et al. (2015), have also reported distinct intervals of RS and RE effects, indicating that not only unattended but even subliminal processing of faces and objects, as well as pure tones, are associated with both increase and decrease of ERPs. It is notable that the same pattern of results have been obtained by Henson et al. (2008), Recasens et al. (2015) and our current study, namely that the RE effects occur later than RS effects.

A limitation of our study is the relatively short stimulus onset asynchrony (SOA) of 570 ms. Due to the short SOA, we cannot fully exclude that the pre‐stimulus 100 ms baseline period contained ERP components from the previous trial. However, it is unlikely that baseline correction (which consists of subtracting a constant) could have significantly contributed to the observed sequence of multiple short intervals of repetition effects.

Scalp‐recorded ERPs result from the linear summation of electric fields generated in the brain; therefore, it is non‐trivial to determine whether changes in a certain ERP component relative to a baseline period were caused by an increase/decrease of a negative/positive potential. Based on our current analysis, we cannot unequivocally disambiguate whether our results are due to RS of negative components or RE of positive components; this ambiguity is inherent to all ERP studies. Nevertheless, several canonical ERP peaks have known timing and scalp distribution. Here, we interpreted amplitude changes during the period of these peaks in the most parsimonious way. For example, a period of decreased ERP overlapping with the early P1 peak is interpreted as RS of a dipolar source activity in the extrastriate cortex projecting its positive field over posterior electrodes (Di Russo, Martinez, Sereno, Pitzalis, & Hillyard, 2002; Murphy, Kelly, Foxe, & Lalor, 2012). While our current results provide objective phenomenological descriptions of repetition effects of scalp‐recorded ERPs, future steps will involve studying repetition effects with more mechanistically interpretable computational and biophysically plausible network models (e.g., Garrido et al., 2009).

## Conflict of interest

We have no conflict of interest or competing interests to disclose.

## Data accessibility

Data are available from the corresponding author upon request.

## Author contributions

GS and IC designed the study. GS, JH, and KES analyzed the data. GS, JH, IC, and KES interpreted the results. All authors contributed to writing the manuscript.

**Abbreviations**
EEG: electroencephalography
ERP: event‐related potential
FWE: family‐wise error
FWHM: full‐width at half‐maximum
GLM: general linear model
PC: predictive coding
PE: prediction error
RE: repetition enhancement
RS: repetition suppression
SPM: statistical parametric mapping
SSA: stimulus specific adaptation

## Supporting information

Video S1: Statistical Parametric Map (SPM) for repetition effects in the post‐stimulus 50‐500 ms interval. Scalp map shows the time‐course of significant clusters (family‐wise error corrected for multiple comparisons at the voxel level (*p* < 0.05 (FWE))), where repetition effects were found with the exponential model. Rectangular panels show time‐course of effects in saggital and coronal planes through the 3D scalp space‐time volume at scalp positions indicated by the blue crosshair in the scalp map panel. Color bar shows F‐values of group statistics.
