# Supplementary material for: Timing of repetition suppression of event‐related potentials to unattended objects
Source: Eur J Neurosci. 2018 Aug 10;52(11):4432–41. doi: 10.1111/ejn.13972 (PMC7818225; doi:10.1111/ejn.13972)
Supplement: Supplementary file 2 [file EJN-52-4432-s002.pdf]

EJN European Journal of Neuroscience FENS Federation of European Neuroscience Societies

Timing of repetition suppression of event-related potentials to unattended objects

Gabor Stefanics, Jakob Heinzle, István Czigler, Elia Valentini and Klaas Enno Stephan

Review timeline:	Submission date:	01 December 2018
	Editorial Decision:	18 February 2018
	Revision Received:	03 April 2018
	Accepted:	16 May 2018

Editor: Alan Pegna
Reviewer 1: Marzia Del Zotto (University Hospitals of Geneva, Switzerland)
Reviewer 2: Ryszard Auksztulewicz (Humboldt-Universität zu Berlin, Germany)

1st Editorial Decision 18 February 2018

Dear Dr. Stefanics,

Your manuscript was reviewed by external reviewers as well as by our Guest Section Editor, Dr. Alan Pegna, and ourselves. The reviews collectively indicate that your experiments generated new and important information. However, there are several issues that need to be clarified/resolved before we can consider your manuscript further for publication in EJN. In particular, both reviewers point out that the manuscript could benefit from a more thorough discussion of the existing literature. Also, one point that must be addressed is the use of a fixed inter-stimulus duration (320ms) and its potential influence on the results.

Please also attend to the following two issues in your revision:
1. Please spell out event-related potentials in the title.
2. The footnote should be removed or relocated to the main text.

Thank you for submitting your work to EJN.

Kind regards,

John Foxe & Paul Bolam
co-Editors in Chief, EJN

Reviews:

Reviewer: 1

Comments to the Author

The manuscript describes an electrophysiological study focused on the intra-trial time course and on the scalp distribution of the repetition effects during visual presentation of black-white drawings representing common objects. They used Statistical Parametric Mapping, a comprehensive analysis framework, to investigate the effects of the stimulus repetition on ERPs over the whole scalp, in a time window of 50-500ms after stimulus onset. Data analyses revealed significant effects due to the:

· RS (repetition suppression) in three consecutive time intervals between 86-140ms, 322-360ms, and 400-446ms, with temporo-parietal, and fronto-temporal distribution, respectively;

· RE (repetition enhancement) between 320 and 340ms over occipito-temporal cortical areas.

They concluded that the neural connectionsof occipital, temporal and frontal cortical regions reflected by RS and RE, underlie the short-term plasticity, required for optimization of perceptual inference, the associated changes in prediction errors (PE) and predictions, over stimulus repetitions during automatic object recognition.

The paper conforms to the definition of Original Research Articles. Authors clearly describe the hypothesis, the study and the methods. They discuss the results and possible implications quite extensively. The manuscript includes all following sessions: Abstract, Introduction, Material and Methods, Results, Discussion.

The language and the grammar are of good quality.

The title clearly and precisely reflects the findings of the paper

The abstract is appropriate and well structured, summarizing perfectly the aim of the study, the results and the conclusions.

The Introduction session is appropriate compared to the context of the research and to the mainaim of the study. However, I kindly ask to explain more extensively the different between this study and the other ones quoted at the end of pg. 3 (e.g. Schendan & Kutas .....Gosling et al.)

The Material and Methods, as well as Results, are well performed. The authors applied a new interesting and valid method of statistical analysis of the EEG signal and ERPs. The number of participants is suitable. However, I have some little remarks:

1. It is not clear to me if the change of the stimulus target (the cross) is always done at the 7th repetition of the same item or it happens randomly.

2. How many repetitions and trials in total there are in each block, please? I kindly require this information in the text.

3. The authors use the word “block” in the text and the word “experiment” in the figure 1B. It would be better to use always the same word to be coherent and immediately understandable.

4. The authors used 60 figures depicting black and white line drawings of common objects. I’d like to know if they are living or non-living entities. If both, are they balanced across categories? Anyway, I kindly ask to write this information clearly in the text, please.

5. The ISI is fixed (320 ms), it does not vary (shortly) in a specific time-frame as usually reported in EEG studies. I kindly ask to justify that choice, please.

6. F-tests have been computed to check significantly ERP differences across the six repetitions (= experimental conditions). However, I don’t understand if the authors used a multiple ANOVA comparing the 6 conditions together (by means of post-hoc comparisons) or they run a T-test, comparing them two by two. I kindly ask to clarify this point, please. However, the significant difference between all 6 conditions is not very clear. For instance, when the authors showed the differences in the figures 2C across repetitions, they presented only 2 conditions (the 1st and the 6th), ...why? If I understood properly, all the conditions are significantly different in the time windows described in the manuscript, except the 2nd and the 4th ones that are similar. I recommend a clarification about this important point that needs to be more detailed in the text and/or in the figures. Eventually, I suggest a table with cross-comparisons of the 6 conditions for each time window.

The Discussion Session adequately addresses the research questions based on the hypothesis posed in the introduction. The conclusions are supported by the data. However, at pg. 8 (line 5 to 8) of the discussion, authors linked each ERP time window to the RS or RE, as follows:

“In the post-stimulus 50-500ms time window. we observed three consecutive intervals of RS in the 86-140ms, 322-360ms, and 400-446ms time window with occipital, temporo-parietal, and fronto-temporal distribution, respectively.”

I suggest that they write the same information in figure 2, thus it is possible to immediately understand which interval is characterized by the RS or RE. Moreover, they can refer to figure in the text as well.

They concluded that repetition effects observed in this study likely reflect automatic perceptual inference operating outside the focus of visual attention, as proposed by theories of perception as unconscious inference. I kindly ask to discuss this point more extensively, please.

Minor comments:

Bibliography:

- 2nd line at pg. 4 in “Stimuli and procedure” session, there is a formatting mistake regarding the citation (no brackets needed in that case)

- Last line at pg. 5 “Flandin and Friston 2017” (reported in the text) vs. 2016 (reported in the bibliography session).

All in all, this paper is a good example of an ERP study about repetition effects, presenting a valid and well performed mass-univariate statistical approach of the entire space*time volume of EEG signal. The authors were able to present this complex topic in an adequately fair and concise manner that is of high quality and impact. I recommend the publication of this manuscript.

Reviewer: 2

Comments to the Author

EJN European Journal of Neuroscience FENS Federation of European Neuroscience Societies

This paper presents the results of an ERP study using repeated visual stimuli. In the analysis, the ERPs to subsequent repetitions of the stimuli are modelled in a GLM using a monotonic parametric regressor coding for stimulus repetition, in order to establish which spatiotemporal clusters of EEG amplitude are modulated by repeated presentation. The findings show that both early and late ERP components are susceptible to repetition suppression (with distinct topographies), and that suppressive effects are followed by repetition enhancement at later latencies. While the paper is generally very well written and the results concisely presented, as the authors will see from the comments below, the paper would benefit from a more thorough discussion of the novelty of the findings.

1. It is understandable that due to space constraints the authors might decide not to report the results of an orthogonal analysis of ERPs to changes of viewing angle. However, the title of the paper and the abstract suggest that the results reported here will pertain to object recognition. On the other hand, the analysis is based on only one type of stimuli (line-drawn objects) with no control conditions. As a result, it is not obvious to what extent the reported results are specific to object recognition, and to what extent they are a replication of previous findings (see also point 3). In this context, it would be interesting to see whether object repetition over different viewing angles leads to more pronounced RS/RE effects.

2. Some of the authors' previous work on MMN and RS in the auditory system (Garrido et al., 2009), also cited in this manuscript, used a similar approach to analyse ERPs but instead of only modelling (monotonic) exponential decay/rise, also included a phasic parametric regressor which was linked to PE precision modulation. What was the reason behind not modelling the biphasic effects on ERP amplitude? Arguably, including other non-monotonic regressors would provide a more thorough characterisation of RS timing – again increasing the impact of the paper.

3. Finally, in the discussion, I missed a section relating one of the central findings – namely that the RE effects are later than the RS effects – to a previous study showing the same pattern of results in the auditory modality (Recasens et al., 2015). In the light of this previous results (and also including other studies cited by the authors), the paper would benefit from a more thorough discussion of the novel aspects of the current study.

4. How did the authors make sure that the baseline (pre-stimulus 100ms) did not contain repetition-related effects? Given the relatively short (and fixed) ISI, the evoked responses to repeated stimuli after baseline correction might be confounded by the amplitude of responses evoked by preceding stimuli. In this respect, the study would have benefitted from introducing jittered ISI and analysing the data using convolution modelling of responses evoked by individual stimuli (see Litvak et al., Neuroimage 2013; 64: 388–98; Spitzer et al., Neuroimage 2015; 129:470–479).

5. In the introduction, second paragraph, it is not immediately clear if the authors list the three mechanisms of short-term plasticity and the three mechanisms of SSA as conceptually related to one another, or as studied independently. Furthermore, adding a brief discussion of NMDA-dependent vs. cholinergic modulatory effects mediating short-term plasticity and SSA would be fitting in the context of predictive coding and precision modulation.

Authors' Response 03 April 2018

Response to comments
Thank you very much for your valuable and helpful comments. Our responses to the comments are listed below. All corrections in the revised manuscript are highlighted in blue font.

Editors' comments
1. In particular, both reviewers point out that the manuscript could benefit from a more thorough discussion of the existing literature.
2. Also, one point that must be addressed is the use of a fixed inter-stimulus duration (320ms) and its potential influence on the results.
3. Please spell out event-related potentials in the title.
4. The footnote should be removed or relocated to the main text.

Response
Thank you very much for your helpful comments and instructions.
1. We have addressed this issue following the suggestions of the Reviewers, please see our detailed point-by-point response below.
2. Several studies use a varying ISI to counteract possible entrainment to periodic stimulation, nevertheless, the use of a fixed ISI is not uncommon either (e.g., Eddy et al., 2006; Recasens et al., 2015). One potentially significant drawback of using a jittered ISI is that ERPs might contain prediction error components elicited by the violation of time expectations due to trial-by-trial variations of the ISI. Here, we aimed to study the decay of ERPs thought to reflect (at least partially) prediction errors about object identity, therefore temporal prediction errors were an important confound to avoid and required us to keep the ISI constant.
It is very unlikely that a confound in baseline correction due to the previous responses affected our results. This is because during baseline correction a constant is subtracted from the whole epoch, i.e., the baseline correction procedure uniformly affected all data points in the analyzed 50-500ms interval. Therefore any effect caused by a confound in baseline correction should have appeared over the whole 50-500ms interval at potentially affected sites. However, we obtained no such a result. We found significant clusters of scalp time-points with durations varying between 20 ms to 54 ms, which is inconsistent with a potential confound due to a 'contaminated' baseline period. Inspecting ERP waveforms in Figure 3C shows that traces for the 1st and 6th presentations are very similar for most datapoints in the 50-500ms epoch, and they only differ in some short time windows which were revealed by statistical parametric mapping.

Nevertheless, we have added the following paragraph to the Discussion: “A limitation of our study is the relatively short stimulus onset asynchrony (SOA) of 570ms. Due to the short SOA, we cannot fully exclude that the pre-stimulus 100ms baseline period contained ERP components from the previous trial. However, it is unlikely that baseline correction (which consists of subtracting a constant) could have significantly contributed to the observed sequence of multiple short intervals of repetition effects.”
3. Done.
4. We have moved the footnote to the main text.
Reviewer #1
The manuscript describes an electrophysiological study focused on the intra-trial time course and on the scalp distribution of the repetition effects during visual presentation of black-white drawings representing common objects. They used Statistical Parametric Mapping, a comprehensive analysis framework, to investigate the effects of the stimulus repetition on ERPs over the whole scalp, in a time window of 50-500ms after stimulus onset. Data analyses revealed significant effects due to the:
• RS (repetition suppression) in three consecutive time intervals between 86-140ms,
322-360ms, and 400-446ms, with temporo-parietal, and fronto-temporal distribution, respectively;
• RE (repetition enhancement) between 320 and 340ms over occipito-temporal cortical areas.
They concluded that the neural connections of occipital, temporal and frontal cortical regions reflected by RS and RE, underlie the short-term plasticity, required for optimization of perceptual inference, the associated changes in prediction errors (PE) and predictions, over stimulus repetitions during automatic object recognition.

The paper conforms to the definition of Original Research Articles. Authors clearly describe the hypothesis, the study and the methods. They discuss the results and possible implications quite extensively. The manuscript includes all following sessions: Abstract, Introduction, Material and Methods, Results, Discussion.
The language and the grammar are of good quality.
The title clearly and precisely reflects the findings of the paper. The abstract is appropriate and well structured, summarizing perfectly the aim of the study, the results and the conclusions.

The Introduction session is appropriate compared to the context of the research and to the main aim of the study. However, I kindly ask to explain more extensively the different between this study and the other ones quoted at the end of pg. 3 (e.g. Schendan & Kutas.....Gosling et al.)

Response
Thank you very much for your positive and encouraging remarks.
Following your suggestion, we have now elaborated more on the difference between our current and previous studies and modified the last paragraph of the Introduction on pg. 4 as follows:
“While studies on repetition of faces and words are relatively abundant (for a recent review, see Schweinberger & Neumann, 2016), there are few electroencephalography (EEG) studies where time-course and topographic distribution of repetition effects to objects were investigated at the whole-scalp level. Typically, most studies focused the analysis on a preselected set of electrodes and confined amplitude measurements to time windows either based on visual inspection of the data or on previous reports in the literature. Furthermore, previous studies often investigated brain responses only to the first repetition relative to the initial presentation, thus ignoring the dynamics of brain responses to further repetitions (Schendan & Kutas, 2003; Henson et al., 2004; Eddy et al., 2006; Gruber et al., 2006; Gruber & Muller, 2006; Guillaume et al., 2009; Gilbert et al., 2010; Kim et al., 2012; Andrade et al., 2015; Gosling et al., 2016). The aim of the current study was to determine the time course and scalp distribution of repetition effects without prior hypotheses about the dynamics (RS vs RE), time course, or scalp distribution of repetition effects. To this end, we analysed the spatio-temporal dynamics of ERPs to black and white line drawings of common objects over six consecutive presentations. We used statistical parametric mapping (SPM; Friston, 2007) to analyse ERP amplitudes at each and every sensor in the poststimulus 50-500ms time window using a mass-univariate approach. Given that most studies focused on the analysis of ERPs to the first repetition only, little is known about the time course of the decay of scalp-recorded ERPs to object stimuli that are repeated multiple times. Therefore we set up three GLMs with parametric regressors incorporating three hypotheses about the time-course of repetition effects. We used an exponential, a linear, and a “change detection” model to identify ERP components that showed a reliable repetition effect and performed Bayesian model comparison (Stephan et al., 2009) in order to identify the model that best explained the observed data.”

The Material and Methods, as well as Results, are well performed. The authors applied a new interesting and valid method of statistical analysis of the EEG signal and ERPs. The number of participants is suitable. However, I have some little remarks:

1. It is not clear to me if the change of the stimulus target (the cross) is always done at
the 7th repetition of the same item or it happens randomly.

Response
The change in the fixation cross occurred randomly, once every 3-6 seconds. Furthermore, trials falling in the 800ms interval following a cross flip were excluded from the analysis; this information had previously been missing in the text. We modified the text on pg. 5 as follows: “Pseudo-randomly every 3-6 seconds, the fixation cross became wider or longer (Fig. 1A). The participants’ task was a speeded button-press to the changes of the cross and reaction time (RT) was recorded. Trials occurring within an 800 ms interval after a change in the fixation cross were excluded from the analysis.”

2. How many repetitions and trials in total there are in each block, please? I kindly require this information in the text.

Response
Thank you for pointing out that this was missing from the Methods. 60 objects were presented in each block, and each object was repeated 8-12 times, before another object was presented. We have added this information to the text on pg. 4, as well as information about the number of trials used for calculating the ERPs that entered the GLM. Additionally, complying with the Editor's request, we have moved the footnote to the text. We now write on pg. 4:
"In each of the four blocks, we recorded event-related potentials (ERP) to 60 black and white line drawings of common objects taken from the picture inventory by Szekely et al. (2004). The 60 object pictures were selected from the following semantic categories: small artifacts (n=39, e.g., book, flag); large artifacts (n=1, bed); objects found in nature (n=2, flower, leaf); things to wear (n=5, e.g., coat, hat); body parts (n=2, feather, heart); foods (n=11, e.g., apple, mushroom). Stimuli were organized into microsequences of 6-10 presentations of an object followed by a close-up or wider-angle view of the same object, and an additional repetition of the same object with its original viewing angle another two times (Figure 1A). ERPs to changes of viewing angle were not analysed here. They were included to study boundary extension effects (e.g., Czigler et al., 2013) and will be published elsewhere. Thus the length of microsequences varied pseudo-randomly between 9-13 presentations. […] To avoid other potential artefacts, epochs with values exceeding ±75 µV on any EEG or EOG channel were rejected from the analysis using the open source software EEGLAB (RRID: SCR_007292, (Delorme and Makeig, 2004)) in the Matlab development environment (MathWorks, Natick, USA). After artifact rejection, the total number of trials (summed over the four blocks) used for calculating the mean ERPs that entered the GLM was 197 (sd=11), 184 (sd=13), 183 (sd=11), 182 (sd=12), 187 (sd=11), 178 (sd=11), for 1st, 2nd, 3rd, 4th, 5th, and 6th presentation of stimuli, respectively."

3. The authors use the word "block" in the text and the word "experiment" in the figure 1B. It would be better to use always the same word to be coherent and immediately understandable.

Response
Thank you. We have removed the word "experiment" from the legend of figure 1B.

4. The authors used 60 figures depicting black and white line drawings of common objects. I'd like to know if they are living or non-living entities. If both, are they balanced across categories? Anyway, I kindly ask to write this information clearly in the text, please.

Response
This is an important point, and we understand your concern. For some types of analyses, potential differences in object categories could certainly be important. However, we do not think that this issue is critically relevant for the analysis presented in this paper. This is because none of our analyses compares responses between different categories. Instead, we focus on the analysis of changes during repeated presentations for each object, irrespective of its semantic category, and demonstrate how, when and where on the scalp event-related brain potentials decay during stimulus repetition. In other words, a comparison of RS/RE between semantic categories is not the topic of the current study.
As mentioned above, the 60 object pictures were selected from the following semantic categories: small artifacts (n=39, e.g., book, flag); large artifacts (n=1, bed); objects found in nature (n=2, flower, leaf); things to wear (n=5, e.g., coat, hat); body parts (n=2, feather, heart); foods (n=11, e.g., apple, mushroom). This information was added to the Methods on pg. 4:
"In each of the four blocks, we recorded event-related potentials (ERP) to 60 black and white line drawings of common objects taken from the picture inventory by Szekely et al. (2004). The 60 object pictures were selected from the following semantic categories: small artifacts (n=39, e.g., book, flag); large artifacts (n=1, bed); objects found in nature (n=2, flower, leaf); things to wear (n=5, e.g., coat, hat); body parts (n=2, feather, heart); foods (n=11, e.g., apple, mushroom). Stimuli were organized into microsequences of 6-10 presentations of an object followed by a close-up or wider-angle view of the same object, and an additional repetition of the same object with its original viewing angle another two times (Figure 1A)."

5. The ISI is fixed (320 ms), it does not vary (shortly) in a specific time-frame as usually reported in EEG studies. I kindly ask to justify that choice, please.

Response
You are absolutely right, many studies have used a varying ISI to counteract possible entrainment to periodic stimulation, nevertheless, the use of a fixed ISI is not uncommon either (e.g., Eddy et al., 2006; Recasens et al., 2015). One potentially significant drawback of using a jittered ISI is that ERPs might contain prediction error components elicited by the violation of time expectations due to trial-by-trial variations of the ISI. Here, we aimed to study the decay of ERPs thought to reflect (at least partially) prediction errors about object identity, therefore temporal prediction errors were an important confound to avoid and required us to keep the ISI constant. Nevertheless, as this point was also raised by Reviewer 2, we have added a comment on this issue in the discussion on pg. 10: "A limitation of our study is the relatively short stimulus onset asynchrony (SOA) of 570ms. Due to the short SOA, we cannot fully exclude that the pre-stimulus 100ms baseline period contained ERP components from the previous trial. However, it is unlikely that baseline correction (which consists of subtracting a constant) could have significantly contributed to the observed sequence of multiple short intervals of repetition effects."

6. F-tests have been computed to check significantly ERP differences across the six repetitions (= experimental conditions). However, I don't understand if the authors used a multiple ANOVA comparing the 6 conditions together (by means of post-hoc comparisons) or they run a T-test,

EJN European Journal of Neuroscience FENS Federation of European Neuroscience Societies

comparing them two by two. I kindly ask to clarify this point, please. However, the significant difference between all 6 conditions is not very clear. For instance, when the authors showed the differences in the figures 2C across repetitions, they presented only 2 conditions (the 1st and the 6th), ...why? If I understood properly, all the conditions are significantly different in the time windows described in the manuscript, except the 2nd and the 4th ones that are similar. I recommend a clarification about this important point that needs to be more detailed in the text and/or in the figures. Eventually, I suggest a table with cross- comparisons of the 6 conditions for each time window.

Response

Thank you for raising this point. As a 2nd –level statistics (at the group level), we computed F-tests to find scalp time-points where mean ERPs were significantly modulated by repeated stimulus presentations. These tests were carried out on beta parameter estimates obtained in the 1st –level statistics involving multiple regression, which in turn were carried out on data of individual participants. These beta estimates quantify the contribution (weight) of the "decay" regressor to the GLM fit at each data point in the scalp space-time volume for each subject. Thus, we did not use an ANOVA to compare the 6 conditions. Instead, we estimated for each participant a GLM with four main regressors corresponding to the four blocks, and a generic decay/rise function as a parametric modulator for each stimulus presentation in each block. The resulting beta estimates were then analyzed at the group level with F-tests. This standard "two-stage summary statistics" approach (Friston et al. 2005; Mumford & Nichols 2009) allowed us to search for scalp time-points where brain potentials showed a decay-like behavior over repeated stimulus presentations without having to constrain our analysis to predefined time windows and EEG channels. This GLM-based analysis yielded scalp-time clusters of voxels that exhibited a significant relation ("activations") with the exponential (or linear, or "change detection") regressor.

In Figure3A (previous Figure 2) we show the numbers of these activations as indicated in Table 1. We did not plot activation #4 separately because it showed a similar dynamics and temporal topography as activation #2 peaking at 346ms. As explained above, these "activations" refer to significant clusters of scalp time-space voxels, where our exponential "decay" regressor showed a significant relation with the ERP data.

In Figure 3C we only show ERPs corresponding to the 1st and the 6th presentations, because showing more superimposed waveforms together with their confidence intervals would make the figure too crowded and impossible to disentangle the traces. To bring this to the attention of the readers, we have added the following information to the caption of Figure 2:

"C) Grand mean ERP waveforms elicited by the 1st and 6th stimulus presentation with 95% confidence interval are shown for illustration purposes. Note that statistics were carried out on 3D scalp space-time parameter estimates which were based on ERPs for the 1st, 2nd, 3rd, 4th, 5th, and 6th stimulus presentation." In Figure 2B, however, we show the scalp ERP data in time windows and at scalp locations (channels) where our statistical parametric maps showed significant repetition effects.

We described the details of the analysis on pg. 5 in the paragraph "Space × time SPM analysis" in the Methods section. To clarify what the results at the group level represent, we have added the following sentence to this paragraph:

"For example, a decrease of a positive ERP component would show a positive correlation with our regressor across repetitions, whereas an increase of a positive component would show a negative correlation with our regressor (conversely for negative components). The estimated regression coefficients (beta parameter estimates) of the decay function for each scalp time-point for each participant were analysed at the group level for all three models (exponential, linear, "change detection"), using a standard two-stage summary statistics approach (Friston et al. 2005; Mumford & Nichols 2009). These parameter estimates represent the relationship (similarity) between the dynamics of ERPs over repeated stimulus presentations and the parametric decay regressors."

The Discussion Session adequately addresses the research questions based on the hypothesis posed in the introduction. The conclusions are supported by the data. However, at pg. 8 (line 5 to 8) of the discussion, authors linked each ERP time window to the RS or RE, as follows:

"In the post-stimulus 50-500ms time window. we observed three consecutive intervals of RS in the 86-140ms, 322-360ms, and 400-446ms time window with occipital, temporo-parietal, and fronto-temporal distribution, respectively."

I suggest that they write the same information in figure 2, thus it is possible to immediately understand which interval is characterized by the RS or RE. Moreover, they can refer to figure in the text as well.

Response

Thank you for suggesting this. We have modified the caption of Figure3A as follows: "Numbers show activations as indicated in Table 2. Panels from top to bottom show observed intervals of RS in the 86-140ms, RE in the 320-340ms, and RS in the 322-360ms and 400-446ms time windows, with occipital, occipito-temporal, temporo-parietal, and fronto-temporal distribution, respectively. Note that activation #4 is not plotted separately as it showed a similar dynamics and temporal topography to that of activation #2 peaking at 346ms." Furthermore, we have added a reference in the Discussion to the Figure: "We observed three consecutive intervals of RS in the 86-140ms, 322-360ms, and 400-446ms time window with occipital, temporo-parietal, and fronto-temporal distribution (Figure 3A), respectively."

They concluded that repetition effects observed in this study likely reflect automatic perceptual inference operating outside the focus of visual attention, as proposed by theories of perception as unconscious inference. I kindly ask to discuss this point more extensively, please.

Response

Thank you for suggesting this. We have added the following text to the Discussion on pg. 9: "This suggests that repetition effects observed in our study likely reflect automatic perceptual inference operating outside the focus of visual attention. Theories of perception as unconscious inference originate from Helmholtz's classical idea that perceptual experience is the "conclusion" of unconscious inductive inference from sensory input (Hatfield, 2002; Kiefer, 2017). In current theories of cortical information processing such as predictive coding (Friston, 2005), RS is

viewed as the result of a process during which the brain minimizes the prediction error (the difference between the predicted and the actual input) with increasing efficacy (due to updating of predictions and the associated synaptic plasticity of cortical connections) during repeated presentations of the same stimulus type. This decrease of prediction errors during processing of a given stimulus, and the change in efficacy of stimulus processing via plasticity in underlying neural circuits corresponds to perceptual inference and learning, respectively (Baldeweg, 2007)."

Minor comments:
Bibliography:

- 2nd line at pg. 4 in "Stimuli and procedure" session, there is a formatting mistake regarding the citation (no brackets needed in that case)
- Last line at pg. 5 "Flandin and Friston 2017" (reported in the text) vs. 2016 (reported in the bibliography session).

Response
Thank you, we have corrected these errors.

All in all, this paper is a good example of an ERP study about repetition effects, presenting a valid and well performed mass-univariate statistical approach of the entire space*time volume of EEG signal. The authors were able to present this complex topic in an adequately fair and concise manner that is of high quality and impact. I recommend the publication of this manuscript.

Response
Thank you for your positive recommendation and constructive criticisms.

Reviewer #2
This paper presents the results of an ERP study using repeated visual stimuli. In the analysis, the ERPs to subsequent repetitions of the stimuli are modelled in a GLM using a monotonic parametric regressor coding for stimulus repetition, in order to establish which spatiotemporal clusters of EEG amplitude are modulated by repeated presentation. The findings show that both early and late ERP components are susceptible to repetition suppression (with distinct topographies), and that suppressive effects are followed by repetition enhancement at later latencies. While the paper is generally very well written and the results concisely presented, as the authors will see from the comments below, the paper would benefit from a more thorough discussion of the novelty of the findings.
Response
Thank you for your constructive criticisms.
1. It is understandable that due to space constraints the authors might decide not to report the results of an orthogonal analysis of ERPs to changes of viewing angle. However, the title of the paper and the abstract suggest that the results reported here will pertain to object recognition. On the other hand, the analysis is based on only one type of stimuli (line-drawn objects) with no control conditions. As a result, it is not obvious to what extent the reported results are specific to object recognition, and to what extent they are a replication of previous findings (see also point 3). In this context, it would be interesting to see whether object repetition over different viewing angles leads to more pronounced RS/RE effects.
Response
Thank you for pointing this out. We realized that the footnote in the previous version of the manuscript might have been ambiguous. In the main text, however, we mentioned that "changes in viewing angle" meant a closer or more distant view of the same object and from the same position. This would have more adequately been described as "changes in visual angle" and essentially meant a slightly smaller or larger picture of the same object, without a change in viewing position. After the change in visual angle, the object was repeated with its original visual angle for two times before another object was presented, thus our current dataset is not suitable to study whether object repetition over different viewing angles affects RS/RE.
We understand your general concern regarding to what degree our results pertain to object recognition. Strictly speaking, the objects in our experiment were task-irrelevant and we cannot assess to what degree they were "recognized". To avoid any confusion or misinterpretation, we removed references to object recognition from the abstract and modified it as follows:
"Current theories of object perception emphasize the automatic nature of perceptual inference. Repetition suppression (RS), the successive decrease of brain responses… These sequential intervals of RS and RE might reflect the short-term plasticity required for optimization of perceptual inference and the associated changes in prediction errors (PE) and predictions, respectively, over stimulus repetitions during automatic object processing."
Furthermore, we changed the Discussion on pg. 9 as follows: "Our findings represent an important advance in the understanding of the time course and scalp distribution of repetition effects in ERP correlates of object perception as they are based on an unbiased mass-univariate statistical approach that covers the entire space□time volume of EEG signals."

2. Some of the authors' previous work on MMN and RS in the auditory system (Garrido et al., 2009), also cited in this manuscript, used a similar approach to analyse ERPs but instead of only modelling (monotonic) exponential decay/rise, also included a phasic parametric regressor which was linked to PE precision modulation. What was the reason behind not modelling the biphasic effects on ERP amplitude? Arguably, including other non-monotonic regressors would provide a more thorough characterisation of RS timing – again increasing the impact of the paper.
Response
Thank you for bringing this to our attention. Garrido et al. (2009) used dynamic causal modeling (DCM) with two temporal basis functions to model changes across stimulus repetitions: a monotonic exponential decay (as in our previous analysis), and a gamma function. They found a monotonic exponential decay for extrinsic connections, while only "intrinsic connections" (postsynaptic gain parameters) showed a biphasic response. The latter appeared to be a fairly subtle effect, and we were not sure how easily it would replicate. However, in response to your query, we have now adopted the same approach as Garrido et al. by including a gamma function as an additional parametric modulator in the

design matrix.
In order to compare the models (exponential-only and the exponential-and-gamma GLMs) formally, we used the Bayesian Information Criterion (BIC) (Schwarz, 1978) approximation to the log model evidence (LME), separately for each participant. Under Gaussian noise (as assumed by the GLM), this leads to an approximation that is a function of the residual sum of squares (RSS):

$$LME \simeq -1/2\ n \ln(RSS/n) - 1/2\ k \ln(n) \qquad (1)$$

where n is the number of data points and k is the number of parameters estimated by the model. Notably, in our case, k is different (8 and 12 for the original exponential-only and the exponential-and-gamma GLMs, respectively).
In order to perform model comparison at the group level, we computed the logarithm of the group Bayes factor (GBF; Stephan et al., 2007) for each voxel, i.e., the sum of ΔΔLME (between models) across subjects. This corresponds to a fixed effects group-level Bayesian model selection (BMS; Stephan et al., 2009) procedure and was done both within a functionally defined mask (of voxels showing repetition effects under either models) as well as on all voxels in the 3D space-time image volume (to perform an unrestricted comparison). The mask comprised all voxels from the SPM analyses where both models (“logical AND” conjunction) had yielded a significant whole-brain corrected effect. We then used a non-parametric Wilcoxon signed rank test to assess the null hypothesis of zero median for ΔLME across all voxels. The results of these analyses are presented for the Reviewer in Figure R1.

Figure R1. Left panel: histogram of ΔLME in voxels where significant repetition effects were observed under the original exponential GLM and the exponential-and-gamma GLM. The distribution shows a clear advantage for the original exponential model (95.36% of voxels > 0). Right panel: ΔLMEs within the whole 3D space-time volume showed similar results (77.63% of voxels > 0).
To characterize the distribution of ΔLME values more formally, we performed null hypothesis testing. An initial one-sample Kolmogorov-Smirnov test indicated that the distributions of ΔLME for voxels within our functionally defined mask (D=0.95, p<10-5) as well as for the whole 3D space-time volume (D=0.74, p<10-5) were not Gaussian. A non-parametric Wilcoxon signed rank test was used to test the null hypothesis of zero median for the ΔLME. The results showed that the median ΔLME was significantly different from zero (Z=-81.47, p<10-5) for voxels within the mask, as well as for voxels within the whole volume (Z=-248.78, p<10-5). Together, these results strongly support the original exponential-only model over the more complex exponential-and-gamma model.
We have not included these new results in the paper since we think that the exponential-and-gamma model is a relatively specific case that has not been considered widely in studies on repetition suppression. (However, we would be very happy to add these results if you consider them important and informative – just let us know.) Furthermore, we realised that a comparison to other alternative models that are more frequently considered for studying repetition effects might be more meaningful, and we are now reporting these additional analyses in the manuscript. Specifically, we compared our original exponential model to two alternative time-courses of repetition effects assuming either: i) a categorical decay (i.e., object 1st presentation > presentations 2nd–6th), or ii) linear decrease. The categorical decay corresponds to a “change detection” model, i.e., it represents the hypothesis that whenever a new object is presented in a sequence, it elicits a phasic response which disappears completely under the following repetitions (e.g., Lieder et al., 2013). While the linear model is not realistic (given that repetition suppression effects must become consecutively smaller in physiological systems with decay mechanisms), we included this regressor as a “null” model, similar to previous studies (Noppeney and Penny, 2006). Thus, we set up another two GLMs, incorporating the hypotheses that repetition effects follow i) a categorical decay or ii) a linear decrease. Accordingly, we have updated Figure 1, and added the design matrices and regressors of “change detection” and linear decrease models.

Figure 1. Paradigm and 1st-level design matrix. A) We used a simple stimulus repetition paradigm where line drawings of everyday objects were repeated 6 to 10 times. Between the 6th-10th presentations a change in the visual angle was introduced, after which the original picture was repeated two times. Note that our analysis focused on the first six presentations where stimuli did not change over repetitions. B) Covariates plotted over the 1st-level design matrix. Image number corresponds to images for mean ERPs to the 1-6 presentations in four experimental blocks (x axis). ERPs were modelled with a parametric modulator and a main regressor for each block (y axis right). The exponential function (mean centred) used for modelling repetition effects for the first experimental block is plotted in blue over the design matrix (y axis left). C and D) Covariates plotted over the 1st-level design matrix for the Change detection and the Linear models, respectively.
We assessed the three models by performing model comparison at the group level as described above. Here, the functionally defined mask was a conjoint mask of voxels showing significant repetition effects under all the three models (i.e., a “logical AND” conjunction. Model comparison showed that the Exponential model was superior compared to the other two models, and that the linear model performed worse than the “change detection” model, both for voxels within the functional mask, as well as for voxels within the whole volume (details are described below). To characterize the distribution of ΔLME values more formally, we performed null hypothesis testing, as described above. The results are summarized in Table 1.
We have added the results of this comparison (exponential, change detection, linear) to the manuscript on pg. 8, as we believe these results represent novel findings in the field. We are not aware of any previous EEG studies investigating stimulus repetition using model comparison across the whole sensor space × time matrix to identify the dynamics of response decay. By comparison, the auditory MMN analysis by Garrido et al. (2009) focused their analysis on activity in four sources (bilateral A1 and STG) and used a different analysis approach (DCM). Our results go beyond classical ERP analyses of repetition effects and illustrate the utility of analysis approaches that investigate the dynamics of repetition effects over multiple stimulus repetitions.
Accordingly, we have extended the Abstract to include information about model comparison as follows:
“Current theories of object perception emphasize the automatic nature of perceptual inference. Repetition suppression (RS), the successive decrease of brain responses to repeated stimuli, is thought to reflect the optimization of perceptual inference through neural plasticity. While functional imaging studies revealed brain regions that show suppressed responses to the repeated presentation of an object, little is known about the intra-trial time course of repetition effects to everyday objects. Here we used event-related potentials (ERP) to task-irrelevant line-drawn objects, while participants engaged in a distractor task. We quantified changes in ERPs over repetitions using three general linear models (GLM) that modelled RS by an exponential, linear, or categorical “change detection” function in each subject. Our aim was to select the model

EJN European Journal of Neuroscience FENS Federation of European Neuroscience Societies

with highest evidence and determine the within-trial time-course and scalp distribution of repetition effects using that model. Model comparison revealed the superiority of the exponential model indicating that repetition effects are observable for trials beyond the first repetition. Model parameter estimates revealed a sequence of RS effects in three time windows (86-140ms, 322-360ms, and 400-446ms) and with occipital, temporo-parietal, and fronto-temporal distribution, respectively. An interval of repetition enhancement (RE) was also observed (320-340ms) over occipito-temporal sensors. Our results show that automatic processing of task-irrelevant objects involves multiple intervals of RS with distinct scalp topographies. These sequential intervals of RS and RE might reflect the short-term plasticity required for optimization of perceptual inference and the associated changes in prediction errors (PE) and predictions, respectively, over stimulus repetitions during automatic object processing."

The following section was added to the Methods on pg. 6:

"Model comparison

Beside using an exponential function as parametric modulator we explored two alternative models previously considered for studying repetition effects (Noppeney and Penny, 2006; Lieder et al., 2013) and compared them with the exponential model. In particular, we considered repetition effects as 1) categorical decay (i.e., object 1st presentation > presentations 2–6) and 2) linear decrease. The categorical decay corresponds to a "change detection" model, i.e., it represents the hypothesis that whenever a new object is presented in a sequence, it elicits a phasic response which disappears completely under the following repetitions. While the linear model is not realistic (given that repetition suppression effects must become consecutively smaller in physiological systems with decay mechanisms), we included this regressor as a "null" model, similar to Noppeney and Penny (2006). Thus, we set up another two GLMs, incorporating these two hypotheses. The design matrices for a single subject are depicted in Fig. 1C and D, respectively.

In order to compare the models formally, we used the Bayesian Information Criterion (BIC) (Schwarz, 1978) approximation to the log model evidence (LME). Under Gaussian noise (as assumed by the GLM), this leads to an approximation of LME that is a function of the residual sum of squares (RSS):

$$LME \simeq -1/2\ n \ln(RSS/n) - 1/2\ k \ln(n) \qquad (1)$$

where n is the number of data points and k is the number of parameters estimated by the model.

We first computed the LME for each voxel in individual participants. In order to perform model comparison at the group level, we computed the sum of ΔLME (between models) across subjects for each voxel. This is equal to the logarithm of the group Bayes factor (GBF; Stephan et al., 2007) and corresponds to a fixed effects group-level Bayesian model selection (BMS; Stephan et al., 2009) procedure. Group model comparison was done both within a functionally defined mask (of voxels showing repetition effects under all models) as well as on all voxels in the 3D space-time image volume (to perform an unconstrained comparison). The mask comprised all voxels from the SPM analyses where all three models had yielded a significant whole-brain corrected effect (logical "AND" conjunction). We then used a non-parametric Wilcoxon signed rank test to assess the null hypothesis of zero median for ΔLME across all voxels."

The following section was added to the Results on pg. 7:

"Model comparison

We assessed the three models by performing model comparison at the group level as described above. The functionally defined mask was a conjoint mask of voxels showing significant repetition effects under any of the three models (logical "AND" conjunction). Fixed-effects Bayesian model comparison revealed that the Exponential model was clearly superior compared to the other two models, and that the Change detection model performed better than the Linear model, both for voxels within the functional mask (Fig. 2A), as well as for voxels within the whole volume (Fig. 2B).

Figure 2. Histograms of ΔLME. A) Histograms over the voxels within a mask defined by the "logical AND" conjunction of significant voxels under any of the three models, and B) over all voxels in the whole 3D space-time volume.

To characterize the distribution of ΔLME values more formally, we performed null hypothesis testing, as described above. The results are summarized in Table 1.

ΔLME	Median	Mean	Std	% of voxels >0	Kolmogorov-Smirnov test (D)	Kolmogorov-Smirnov test (p)	Wilcoxon signed rank test (Z)	Wilcoxon signed rank test (p)
EXP vs LIN (within mask)	38.77	43.59	14.24	100	1	<0.00001	-53.58	<0.00001
EXP vs CDT (within mask)	16.73	21.48	11.42	100	1	<0.00001	-53.58	<0.00001
CDT vs LIN (within mask)	-21.76	-22.11	9.63	0.57	0.9839	<0.00001	-53.57	<0.00001
EXP vs LIN (all voxels)	25.03	29.15	18.58	98.36	0.9704	<0.00001	-375.75	<0.00001
EXP vs CDT (all voxels)	13.78	13.73	11.03	89.28	0.8439	<0.00001	-348.01	<0.00001
CDT vs LIN (all voxels)	-12.77	-15.43	19.98	21.43	0.7286	<0.00001	-283.30	<0.00001

Table 1. Summary of model comparison statistics. The results show that the distribution of ΔLME values are not Gaussian and median ΔLME values are significantly different from zero for all comparisons. The absolute value of median ΔLME in all comparisons was >12. Notably, a difference in LME >5 is considered as very strong evidence in favour of the superior model (Kass and Raftery, 1995)."

Finally, the following sentence was added to the ERP results section on pg. 8:

ERP results

"Given that model comparison showed that the GLM with the exponential decay function explains the data best, we used the space x time clusters with significant results for the winning model to illustrate repetition effects. We start from scalp topographies and then show using time-windowed data and conventional ERP plots of the effects that lead to significant results in SPM. Scalp topographies of the SPMs are shown in Figure 3A for the cluster maxima."

3. Finally, in the discussion, I missed a section relating one of the central findings – namely that the RE effects are later than the RS effects – to a previous study showing the same pattern of results in the auditory modality (Recasens et al., 2015). In the light of this previous results (and also including other studies cited by the authors), the paper would benefit from a more thorough discussion of the novel aspects of the current study.
Response
Thank you for bringing this up. We were aware of the elegant study by Recasens et al. (2015) and had previously cited it in the Introduction: "Thus, PC offers explanations for the phenomena observed during repeated stimulus presentation: larger responses to unpredicted events, and attenuation as well as enhancement to repeated events (Egner et al., 2010; de Gardelle et al., 2013; Recasens et al., 2015)." Following your suggestion, we have now added the following to the Discussion on pg. 10:
"Repetition paradigms with masked object (Eddy et al., 2006) and face (Henson et al., 2008) stimuli, as well as an auditory study by Recasens et al. (2015), have also reported distinct intervals of RS and RE effects, indicating that not only unattended but even subliminal processing of faces and objects, as well as pure tones, are associated with both increase and decrease of ERPs. It is notable that the same pattern of results have been obtained by Henson et al. (2008), Recasens et al. (2015) and our current study, namely that the RE effects occur later than RS effects."

4. How did the authors make sure that the baseline (pre-stimulus 100ms) did not contain repetition-related effects? Given the relatively short (and fixed) ISI, the evoked responses to repeated stimuli after baseline correction might be confounded by the amplitude of responses evoked by preceding stimuli. In this respect, the study would have benefitted from introducing jittered ISI and analysing the data using convolution modelling of responses evoked by individual stimuli (see Litvak et al., Neuroimage 2013; 64: 388–98; Spitzer et al., Neuroimage 2015; 129:470–479).
Response
You are right that previous studies have often used a varying ISI to counteract possible entrainment to periodic stimulation, nevertheless, the use of a fixed ISI is not uncommon either (e.g., Eddy et al., 2006; Recasens et al., 2015). One potentially significant drawback of using a jittered ISI is that ERPs might contain prediction error components elicited by the violation of time expectations due to trial-by-trial variations of the ISI. Here, we aimed to study the decay of ERPs thought to reflect (at least partially) prediction errors about object identity, therefore temporal prediction errors were an important confound to avoid and required us to keep the ISI constant.
You are also right that a longer SOA would have been beneficial to dispel any potential concerns that the pre-stimulus baseline could be contaminated by repetition effects. However, in our case, it seems unlikely that a confound in baseline correction due to the previous responses affected our results. This is because during baseline correction a constant is subtracted from the whole epoch, i.e., the baseline correction procedure uniformly affected all data points in the analyzed 50-500ms interval. Therefore any effect caused by a confound in baseline correction should have appeared over the whole 50-500ms interval at potentially affected sites. However, we obtained no such a result. We found significant clusters of scalp time-points with durations varying between 20 ms to 54 ms, which is inconsistent with a potential confound due to a 'contaminated' baseline period. Inspecting ERP waveforms in Figure 3C shows that traces for the 1st and 6th presentations are very similar for most datapoints in the 50-500ms epoch, and they only differ in some short time windows which were revealed by statistical parametric mapping.
Nevertheless, we have added the following paragraph to the Discussion on pg. 10:
"A limitation of our study is the relatively short stimulus onset asynchrony (SOA) of 570ms. Due to the short SOA, we cannot fully exclude that the pre-stimulus 100ms baseline period contained ERP components from the previous trial. However, it is unlikely that baseline correction (which consists of subtracting a constant) could have significantly contributed to the observed sequence of multiple short intervals of repetition effects."

5. In the introduction, second paragraph, it is not immediately clear if the authors list the three mechanisms of short-term plasticity and the three mechanisms of SSA as conceptually related to one another, or as studied independently. Furthermore, adding a brief discussion of NMDA-dependent vs. cholinergic modulatory effects mediating short-term plasticity and SSA would be fitting in the context of predictive coding and precision modulation.
Response
Thank you for pointing this out. You are right, it is not clear how exactly the three mechanisms of short-term plasticity in synapses (facilitation, augmentation, and depression) relate to the three mechanisms that contribute to SSA (somatic afterhyperpolarization, synaptic depression due to depletion of presynaptic vesicles, circuit-level mechanisms). The in-depth treatment of this issue is beyond the scope of our current study, therefore, in order to prevent confusion, we have removed the sentence about the three mechanisms of short-term synaptic plasticity from the Introduction. The changed section reads now as follows:
"Repetition-related phenomena in the visual system are complex and likely represent a compound of distinct neural processes, the mechanisms of which are not fully understood yet (Ibbotson, 2005; Grill-Spector et al., 2006; Solomon & Kohn, 2014). At least three mechanisms contribute to SSA, including i) somatic afterhyperpolarization, ii) synaptic depression due to depletion of presynaptic vesicles, and iii) circuit-level mechanisms (Kohn, 2007; von der Behrens et al., 2009)."
Regarding modulatory neurotransmitters that are thought to mediate short-term plasticity and SSA we have added the following section to the Introduction on pg. 3:
"From the perspective of PC, adaptation or RS can be understood as a neural correlate of perceptual learning, i.e. the optimized process of perceptual inference where PEs to repeated stimuli are explained away more efficiently due to synaptic plasticity in sensory circuits (Baldeweg, 2007; Garrido et al., 2009). Conversely, RE could represent increased neural activity during the "sharpening" of predictions corresponding to increasing precision (implemented by increased postsynaptic gain) over repetitions. Encoding precision is important since veridical perception not only rests on the content of sensory signals, but also on the confidence or precision of the signals that drive inference. It has been suggested that the brain might employ mechanisms to encode precision by relying on modulatory neurotransmitters that regulate the gain or

excitability of populations (Friston et al., 2016). Specifically, cholinergic mechanisms might affect the encoding of sensory precision by modulating postsynaptic gain (Feldman and Friston, 2010; Moran et al., 2013). Alternatively, cholinergic mechanisms can also affect the function of N-methyl-D-aspartate (NMDA) receptors (Aramakis et al., 1997; Chen et al., 2008) which, in turn, are thought be involved in signaling both prediction errors and predictions in cortical hierarchies (Friston 2005; Corlett et al. 2011; Stephan et al. 2016). In sum, PC offers potential explanations for the phenomena observed during repeated stimulus presentation: larger responses to unpredicted events, and attenuation as well as enhancement to repeated events (Egner et al., 2010; de Gardelle et al., 2013; Recasens et al., 2015)."

References (newly added)

Aramakis V, Bandrowski A, Ashe J (1997) Activation of muscarinic receptors modulates NMDA receptor-mediated responses in auditory cortex. Exp Brain Res. 113:484-496.
Chen G, Chen P, Tan H, Ma D, Dou F, Feng J, Yan Z (2008) Regulation of the NMDA receptor-mediated synaptic response by acetylcholinesterase inhibitors and its impairment in an animal model of Alzheimer's disease. Neurobiol Aging. 29:1795-1804.
Corlett PR, Honey GD, Krystal JH, Fletcher PC. (2011) Glutamatergic model psychoses: prediction error, learning, and inference. Neuropsychopharmacology. 36:294-315.
Feldman H, Friston KJ (2010) Attention, uncertainty, and free-energy. Front Hum Neurosci. 4:215.
Friston KJ (2007) Statistical parametric mapping. In Friston, K.J., Ashburner, J., Kiebel, S.J., Nichols, T.E., Penny, W.D. (eds) Statistical Parametric Mapping: The Analysis of Functional Brain Images. Academic Press, London. pp. 10-31.
Friston KJ, Brown HR, Siemerkus J, Stephan KE (2016) The dysconnection hypothesis (2016). Schizophr Res. 176:83-94.
Friston KJ, Stephan KE, Lund TE, Morcom A, Kiebel S. (2005) Mixed-effects and fMRI studies. Neuroimage. 24:244-252.
Kass RE, Raftery AE (1995) Bayes Factors. JASA 90:773-795.
Lieder F, Daunizeau J, Garrido MI, Friston KJ, Stephan KE (2013) Modelling Trial-by-Trial Changes in the Mismatch Negativity. PLoS Comput Biol 9(2): e1002911.
Moran RJ, Campo P, Symmonds M, Stephan KE, Dolan RJ, Friston KJ (2013) Free energy, precision and learning: the role of cholinergic neuromodulation. J Neurosci. 33:8227-8236.
Mumford JA, Nichols T. (2009) Simple group fMRI modeling and inference. Neuroimage. 47:1469-1475.
Noppeney U, Penny WD (2006) Two approaches to repetition suppression. Hum Brain Mapp 27:411-416.
Schwarz GE (1978) Estimating the dimension of a model, Annals of Statistics 6:461-464.
Stephan KE, Diaconescu AO, Iglesias S. (2016) Bayesian inference, dysconnectivity and neuromodulation in schizophrenia. Brain. 139(Pt 7):1874-1876.
Stephan KE, Penny WD, Daunizeau J, Moran RJ, Friston KJ (2009) Bayesian model selection for group studies. Neuroimage 46:1004-1017.
Stephan KE, Weiskopf N, Drysdale PM, Robinson PA, Friston KJ (2007) Comparing hemodynamic models with DCM. Neuroimage 38:387-401.
